# Plasma metabolomic signatures for copy number variants and COVID-19 risk loci in Northern Finland populations

**DOI:** 10.1038/s41598-025-94839-9

**Published:** 2025-04-16

**Authors:** Tisham De, Lachlan Coin, Jethro Herberg, Michael R Johnson, Marjo-Riitta Järvelin

**Affiliations:** 1 Department of Epidemiology and Biostatistics, School of Public Health, Imperial College London, London, UK; 2 Department of Genomics of Common Diseases, Imperial College London, London, UK; 3 Department of Infectious Disease, Imperial College London, London, UK; 4 Department of Microbiology and Immunology, Institute for Infection and Immunity, University of Melbourne at The Peter Doherty, Melbourne, Australia; 5 Department of Brain Sciences, Imperial College London, London, UK; 6 Centre for Life Course Health Research, Faculty of Medicine, University of Oulu, Oulu, Finland; 7 Unit of Primary Health Care and Medical Research Center, Oulu University Hospital, Oulu, Finland; 8 Centre for Environment and Health, Imperial College London, London, UK; 9 Biocenter Oulu, University of Oulu, Oulu, Finland

**Keywords:** Genetics, Immunology, Molecular biology

## Abstract

Copy number variants (CNVs) are an important class of genomic variation known to be important for human physiology and diseases. Here we present genome-wide metabolomic signatures for CNVs in two Finnish cohorts—The Northern Finland Birth Cohort 1966 (NFBC 1966) and NFBC 1986. We have analysed and reported CNVs in over 9,300 individuals and characterised their dosage effect (CNV-metabolomic QTL) on 228 plasma lipoproteins and metabolites. We have reported reference (normal physiology) metabolomic signatures for up to ~ 2.6 million COVID-19 GWAS results from the National Institutes of Health (NIH) GRASP database, including for outcomes related to COVID-19 death, severity, and hospitalisation. Furthermore, by analysing two exemplar genes for COVID-19 severity namely LZTFL1 and OAS1, we have reported here two additional candidate genes for COVID-19 severity biology, (1) NFIX, a gene related to viral (adenovirus) replication and hematopoietic stem cells and (2) ACSL1, a known candidate gene for sepsis and bacterial inflammation. Based on our results and current literature we hypothesise that (1) charge imbalance across the cellular membrane between cations (Fe^2+^, Mg^2+^ etc.) and anions (e.g. ROS, hydroxide ion from cellular Fenton reactions, superoxide etc.), (2) iron trafficking within and between different cell types e.g., macrophages and (3) systemic oxidative stress response (e.g. lipid peroxidation mediated inflammation), together could be of relevance in severe COVID-19 cases. To conclude, our unique atlas of univariate and multivariate metabolomic signatures for CNVs (~ 7.2 million signatures) with deep annotations of various multi-omics data sets provide an important reference knowledge base for human metabolism and diseases.

**Supplementary Information:**

The online version contains supplementary material available at 10.1038/s41598-025-94839-9.

## Introduction

Copy-number variation (CNV) is an important class of mutation which affects roughly 9–10% of the human genome sequence. Several landmark studies have profiled CNVs in large disease and multi-ancestry populations and some recent studies have characterised CNVs in longitudinal cohorts^1,2^. Current literature suggests that the functional effects of CNVs remain relevant in neuropsychiatric disorders^3^ and cancer^4–6^ but their full relevance in common heritable diseases or their role in normal human physiology and phenotypes still remains unclear and perhaps underdetermined^7^. In our previous study^8^, we successfully characterised several common CNVs in the TSPAN8 gene and showed that it has a pleiotropic effect on many metabolites. Here, we have extended our previous analysis and report genome-wide autosomal results for CNV analysis in over 9,300 Northern Finland individuals and the quantitative effect of these CNVs on 228 ^1^H NMR characterised lipid and fatty acid phenotypes. Our multimodal association analysis of genomic variations including CNVs, single nucleotide variation (SNP), log R ratio (LRR) using both univariate and multivariate models resulted in a diverse class of metabolomic signatures, which were further enriched by extensive multi-omics annotations. Importantly, as an exemplar analysis, we were able to successfully characterise metabolomic signatures of known COVID-19 GWAS loci in our cohorts and discover novel biology related to iron trafficking (cation-anion exchange or charge imbalance) and macrophages. We note that these results can explain a substantial number of observations from current literature on COVID-19 disease biology for OAS1 and LZTFL1. In summary, our results provide a landscape of CNVs observed in a large bottlenecked Finnish longitudinal cohort and further substantiates its dosage effect on 228 metabolite measurements. This rich resource of metabolic signatures with an extensive multi-omics annotation database is likely to be an important knowledge base for human genetic variation and its effect on diseases.

## Results

### CNV landscape

**Fig. 1:**
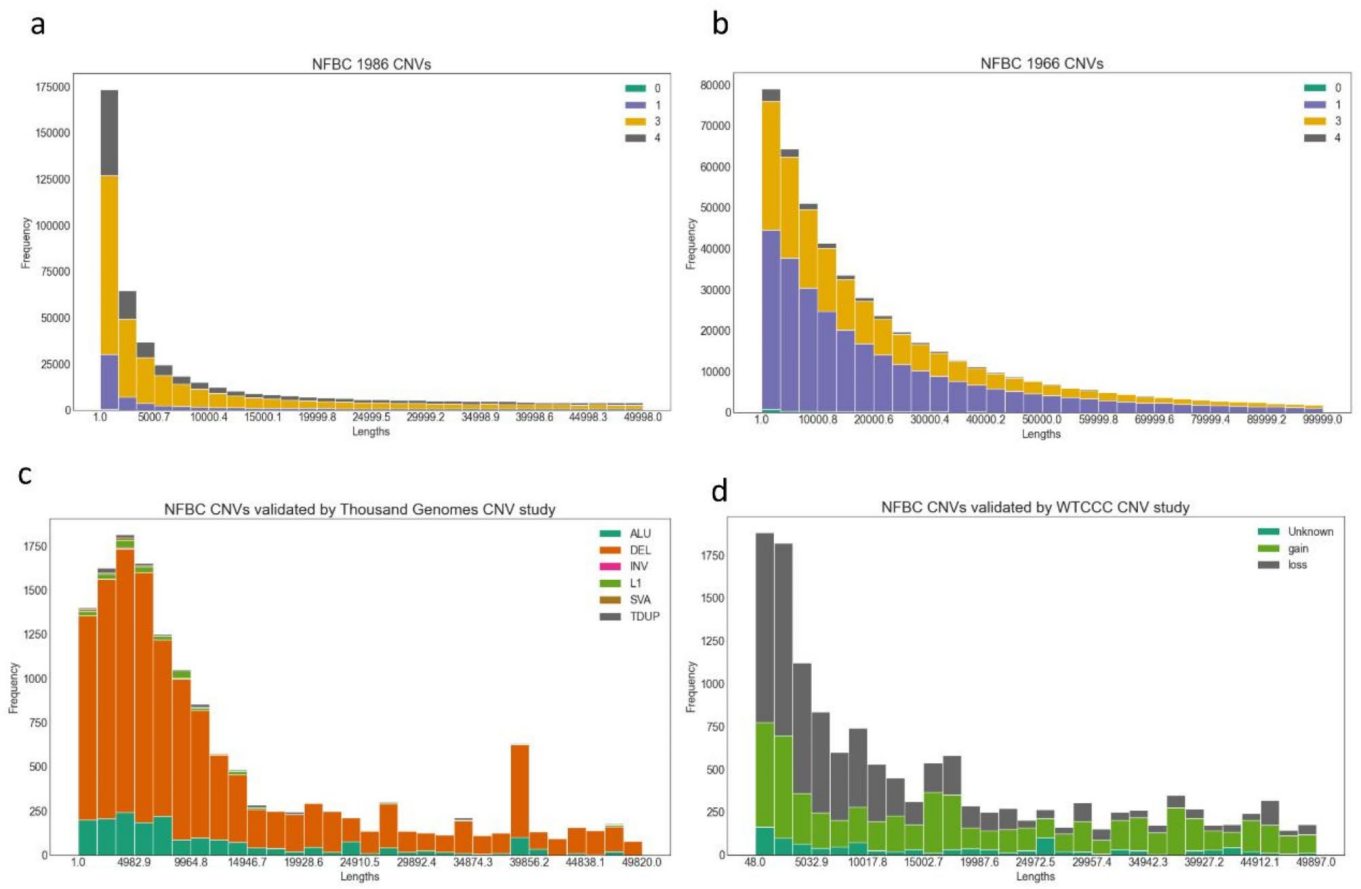
Length distribution of CNVs. X-axis denotes unique lengths of CNV (base pairs) observed in NFBC cohorts. (**a**) NFBC 1986. (**b**) NFBC 1966 (**c**) NFBC CNVs validated by the one thousand genomes study (**d**) NFBC CNVs validated by the WTCCC CNV study. The genomic characteristics of all CNVs is available in the accompanying database.

In NFBC 1986, genotyped on Illumina Cardio-MetaboChip, we detected 863,753 distinct CNV breakpoints, with most overlapping with each other. Out of these, 246 were homozygous deletions (CNV type 0), 84,524 heterozygous deletions (CNV type 1), 550,829 heterozygous duplications (type 3) and 228,154 homozygous duplications (type 4) (Fig. 1a). In NFBC 1966, we detected 510,622 distinct CNV breakpoints with 1751, 296,040, 195,322 and 17,509 counts for CNV types 0,1, 3, and 4 respectively (Fig. 1b). Based on expected CNV genotypes, the most common genic CNV was found to be in the gene OLFML2B (1:161976988, type 3 and 4, MAF = 0.5) for NBFC 1986 and in LRRK2 (12:40601708, type 1, 3 and 4, MAF = 0.447) for NFBC 1966. LRRK2 also harboured the most common meta-analysis CNV for NFBC 1986 and NFBC 1966 (12:40601708, type 1, 3 and 4, MAF = 0.447). As per CNV validation, 21,144 and 18,124 NFBC CNVs (loci) overlapped with WTCCC CNV study and the thousand genomes project (1KGP) CNV results respectively (Fig. 1c and d). Further details of CNV breakpoints, their annotations and lipid signatures are available in an accompanying multi-omics signature database **supplementary Table ****1**. Of note, all reported CNVs here have at least one probe with association p-value ~ 0.05 with one or more of the metabolomic phenotypes.

### Metabolomic signatures

**Fig. 2:**
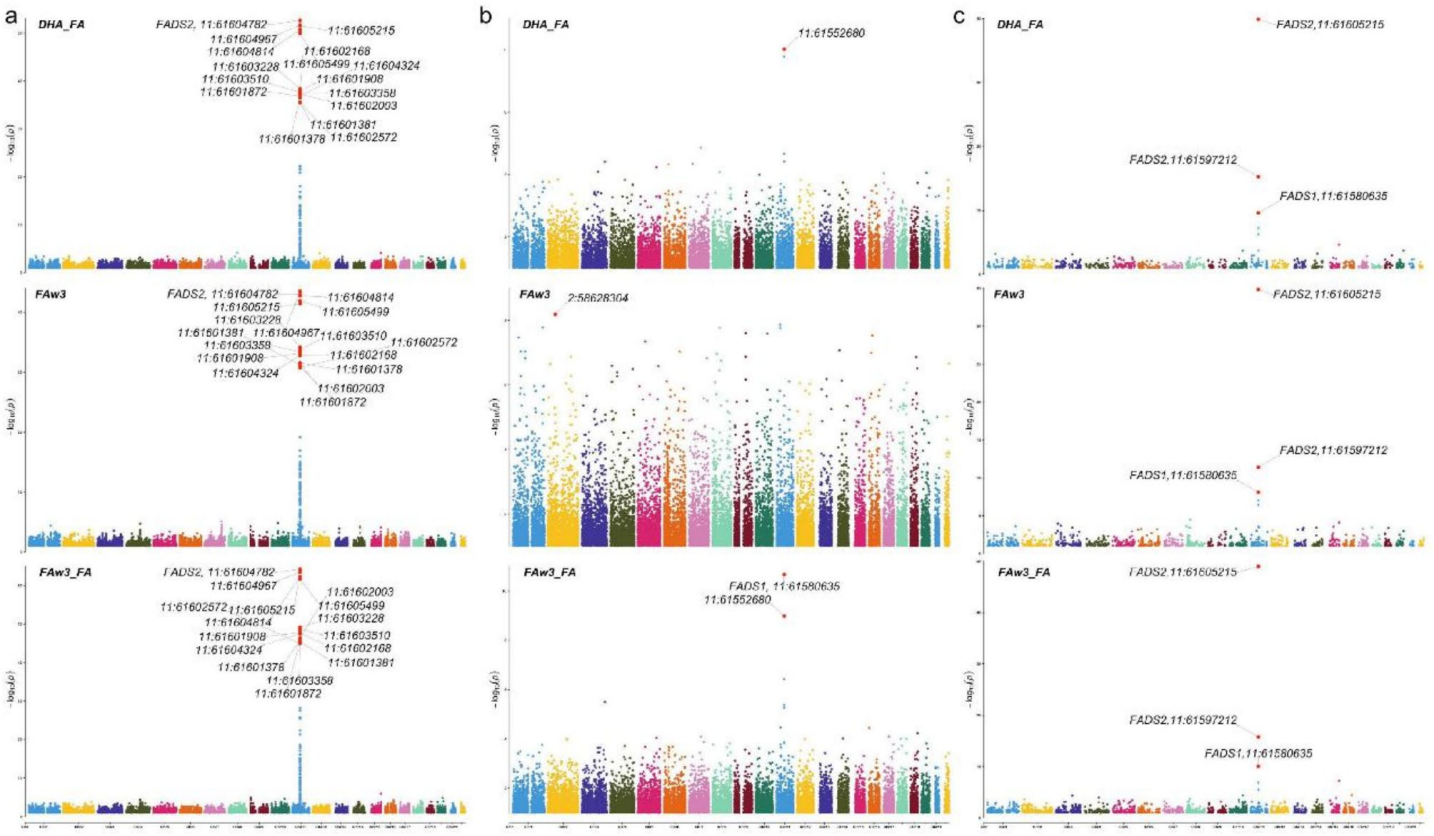
Manhattan plot for CNV associations with the top three metabolomic lipids. (**a**) NFBC 1986 (**b**) NFBC 1966 (**c**) Meta analysis of NFBC 1986 and NFBC 1966.

Based on CNV genotypes and LRR measurements (with 50 LRR pcs as covariates), we generated univariate and multivariate metabolomic signatures from 228 ^1^H NMR metabolites (**Supplementary Table ****2**) for genotyping probes in NFBC 1986 (*n* = 196,475) and for probes in NFBC 1966 (*n* = 382,960) and in addition for common probes (*n* = 10,764) between the two cohorts. The univariate association results consisted of a) ~ 1.93 million association results in NFBC 1986 (Fig. 2a), b) ~ 3.43 million association results in NFBC 1966 (Fig. 2b) c) 328,985 meta-analysis (Fig. 2c) and d) 968,867 pooled association results (**Supplementary Table ****3**). The most significant locus in the meta-analysis was in the FADS2 gene (11:61605215) with p-value 1.06e-49 (*n* = 8,630, MAF = 0.07) for the phenotype FAw3_FA (Ratio of omega-3 fatty acids to total fatty acids) (Fig. 2c). In the pooled analysis, a locus in the LRRK2 gene (12:40601708) was most significant with p-value < 2.225074e-308 for the phenotype Crea (Creatinine). In the multivariate MultiPhen approach for CNV genotypes (all phenotypes used together in a single model) the most significant locus was in the gene CLCN6 (1:11900825) with p-value ~ 2.22e-308 and the most significant multivariate signature (variable selection) was found to be in an intergenic region with p-value ~ 2.22e-308, MAF = 0.164 and a signature consisting of 31 metabolites (**Supplementary Table ****3**). We observed similar trends in CNV pleiotropy across NBFC 1986 and NFBC 1966 (**Supplementary Fig. 1**). Next, we repeated this analysis using only LRR measurements. In total we generated ~ 7.2 million CNV genotype signatures and ~ 6.5 million LRR signatures across all cohorts and approaches. In addition, we have also reported nominal SNP signatures for NFBC 1986 and NFBC 1966 ~ 0.6 million association results. We applied a global filter of p-value ~ 0.05 to all signatures.

### Metabolomic signatures for COVID-19 GWAS loci

**Fig. 3:**
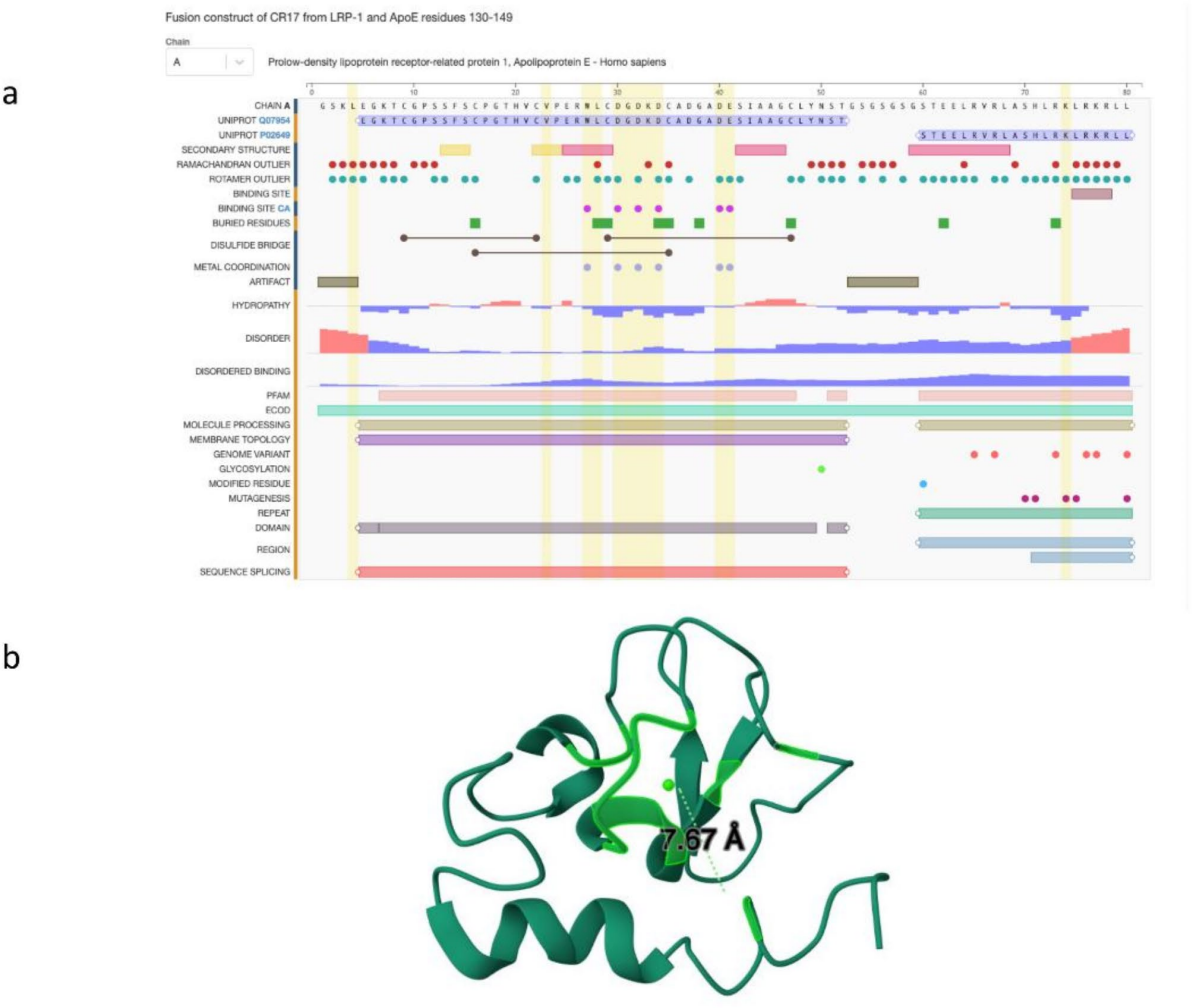
Three-dimensional structure of ApoE fusion protein. Complement repeats (CR) in low-density lipoprotein receptor (LDLR) protein have a high-affinity calcium-binding site allowing them to fold properly. This figure shows ApoE amino acid residues 130–149 interacting with CR17 of LDLR-related protein 1 bound to calcium ion (PDB id 2KNY).

We downloaded UK Biobank GWAS results for COVID-19 from the GRASP database which were divided into categories related to COVID-19 (a) susceptibility (b) death (c) hospitalisation and (d) severity (**Supplementary Table ****4**). In total we obtained 65 GWAS cohorts across all ancestry groups which were further stratified by gender. Next, leveraging genomic location or in a variant (CNV or SNP) agnostic way, we were able to match ~ 2.6 million COVID-19 GWAS results to NFBC 1986 and NFBC 1966 metabolomic signatures at a p-value threshold of ~ 0.05. Across all 65 groups, the top two significant loci were: rank (1) ABO (9:136145425, rs9411378, MAF = 0.22, p-value = 8.6e-11) and rank (2) LZTFL1 (3:45864732, rs10490770, MAF = 0.07, p-value = 5.03e-10). The most significant locus (rank = 1) associated with death in the European population was in the APOE gene (19:45410002, rs769449, MAF = 0.12, p-value = 3.16e-7) (Fig. 3). Of note, allele frequency of rs769449 is variable across different populations with highest frequency in the Finnish population with MAF = 0.16, Non-Finnish European population with MAF = 0.11, East Asian MAF = 0.08 and African/African American MAF = 0.01. We have reported the druggability options for rs769449 in our multi-omics database and further demonstrated an example use case of statin medication for a LDL related gene (**Supplementary Figs. 2**,** 3**). Importantly, as an exemplar case study, we analysed in detail two well-known genes for COVID-19 severity namely (a) LZTFL1 and (b) OAS1 - both reported to have Neanderthal origins^9,10^. The selection of these two genes is based on the results of mapping COVID-19 GWAS results onto NFBC genotyping probes where LZTFL1 was ranked 2 in the overall p-value analysis and was also amongst the top three loci for COVID-19 severity. OAS1 was selected based on its relevance in COVID-19 literature and its metabolomic signatures. We have carried out comprehensive genome-wide signature analysis for both these genes using CNV genotypes and LRR across all datasets and methods. We found a general trend for meta-analysis and pooled analysis of NFBC 1986 and NFBC 1966, where both LZTFL1 and OAS1 consistently had a high signature overlap with NFIX and ACSL1 (**Supplementary Table ****5**). In this signature analysis we also discovered a new gene of interest OASL, an important paralog of OAS1, which is known to be involved in interferon gamma signalling.

### Summary of multi-omics annotations

The beacon section of the multi-omics signature database lists all genomic probes with their location (hg19), variant effect (intron, exon, intergenic etc.) and HGNC gene family (**Supplementary Table ****1**). All probes are annotated with flags (yes = 1, no = 0) for metabolomic signatures, LRR signatures and presence of CNV genotypes in NFBC 1986 or NFBC 1966. CNVs are validated by the WTCCC CNV study results (21,144 loci validated) and thousand genomes CNV results (18,124 loci validated). Metabolomic signatures are validated with annotations from the global lipids consortium database (1,867 loci validated). In addition, the beacon section also has numerous flags in order to look up other multi-omics data present in the database (e.g. GWAS catalogue, OMIM, druggability etc.). Some highlights of other data tables in the database include DrugBank (https://go.drugbank.com/) information for up to 24,380 metabolomic signatures, ~ 2 million matching eQTL signals from the GTex database, reference metabolomic signature for ~ 14,000 OMIM/Orphanet mendelian disease genes, 37,862 loci overlapping with a known cancer driver mutation (in a 4 bp window) and importantly, a fully annotated database table for up to ~ 2.6 million GWAS results for UK Biobank COVID-19 analysis, stratified by ancestry and gender.

## Discussion

CNVs remain relevant in current literature and are often reported in human genetic studies related to rare diseases, cancer and neuropsychiatric disorders. Due to the high deleterious effect of CNVs on human phenotypes it is important to explore their effect on intermediate phenotypes such as lipids, fatty acids and various classes of metabolites. Recently several SNP based metabolomic QTL studies were successful in demonstrating that^1^H NMR based metabolomic measurements from a single drop of blood can prove to be a powerful tool for simultaneously predicting morbidity and many common diseases^11,12^. However, at present there are relatively few studies with large sample sizes analysing the dosage effect of CNVs on the normal human metabolome. Our study here addresses this important knowledge gap in current literature and further leverages these results to uncover an important piece of biology related to COVID-19 severity (further discussed below). Some comparable studies to ours are the recently published metabolome QTL analysis of the UK and Japan Biobanks. Here, we highlight some distinguishing features of our study. First, our discovery and replication cohorts are from a bottlenecked and linguistically isolated population from Northern Finland, hence, less likely to be confounded by population structure or phenotypic heterogeneity. Second, unlike the UK Biobank study where there was a minimum of four-hour gap between last meal and blood collection, in the NFBC study all participants were subject to overnight fasting. A CNV metabolomic QTL analysis of the UK Biobank has not been reported so far but may be expected in future data releases. Such datasets will provide an important benchmark for comparing reference metabolism in humans. In addition, it will further aid in the interpretation and fine-mapping of genomic loci with a-priori disease hypothesis from the large compendium of current GWAS studies. In the long term such biomarker related information could be very useful for population level healthcare practices related to prognosis of common diseases, ageing, physiology (BMI, height) and lifestyle-based outcomes such as educational attainment, medication use, diet etc. Our comprehensive set of metabolomic signatures covers several different modalities of genomic variation including CNVs, log R ratio and SNPs. In addition, each class of genomic variation is further analysed through univariate and multivariate association models at a p-value resolution of 0.05. The accompanying multi-omics annotation database further provides genomic and disease specific context for all reported metabolomic signatures and contains a beacon table which provides comprehensive cross-referencing of all results. The database could potentially be updated and integrated with other datasets in future thus providing new insights and understanding with time. As an exemplar case study of the application of our metabolomic signatures, here, we discuss in detail the cellular biology of two well-known genes for COVID-19 severity namely LZTFL1 and OAS1. Both these genes have Neanderthal origins and were reported to be strongly associated with severe COVID-19 by several independent studies^9,10^. In our genome wide signature comparison analysis, we found LZTFL1 to be closely related to NFIX and OAS1 to be linked to both NFIX and ACSL1. Next, based on current literature, we elucidate how these genes relate to the biology of severe COVID-19.

First, we make important observations regarding the role of oxylipins in resident macrophages, ACSL1 and other members of the ACSL gene family in bacterial inflammation cases of neonatal sepsis^13,14^. Current literature suggests that there are several different isoforms of the ACSL gene family with varied expression and function in different tissues^15–17^. For instance, ACSL1 and ACSL4 isoforms are active in the liver but ACSL3 and ACSL6 isoforms are predominantly active in the brain. We highlight five members of this gene family (ACSL1,3–6) have a common function of transporting oxylipins into the mitochondria and further note that ACSL1 and ACSL4 but not ACSL6 are inhibited by Triacsin C, an inhibitor of long chain fatty acyl-CoA synthetase. Of note, ACSL1 and ACSL4 can affect ferroptosis (iron-dependent cell death) through independent mechanisms^18–21^. A recent study also explains the role of ACSL3 and ACSL4 in iron related oxidative cell injury and cell death due to lipid peroxidation of polyunsaturated fatty acids (PUFAs)^22^. In immunometabolism, oxylipins, along with other classes of oxysterols such as the CH25H are known to activate immune cells such as macrophages^23^ and B cells. Such cascades of immune activation, which is essentially a countermeasure to stop lipid peroxidation^24^ (or free radical propagation) across the body and prevent oxidative tissue damage, however in consequence can lead to inflammation or formation of plaques, as often seen in multiple tissues such as blood vessels, arteries and brain cells^25^. We postulate that such inflammation mediated tissue degeneration, if indeed operative in COVID-19, could be an explanation for symptoms related to hyperinflammation^26^ and multi-organ failures seen in severe cases^27^.

Next, we highlight results from two landmark studies related to COVID-19 namely the GWAS analysis of severe COVID-19^27^ and the COVID-19 host genetics initiative^29,30^. In Fig. 2 of the first study^28^, we note that OAS1 and ACSL6 had significant change in protein concentration in the generalised summary-level data Mendelian randomization (GSMR) results for RNA expression analysis. This GSMR result possibly suggests that there is a switch of cellular function from OAS1-ACSL1 pathways in healthy individuals, as characterised by our metabolomic signatures in NFBC cohorts to OAS1-ACSL6 related severity pathways in COVID-19. Further, based on results from this study and the COVID-19 host genetics initiative and its update, we further highlight several other genes of interest for our COVID-19 severity hypothesis, namely BCL11A, SLC22A31, SLC6A20, SLC2A5 and HBBP1. Here, we illuminate our hypothesis further considering these genes. First we note that both NFIX^31^ and BCL11A^32^ (a distal regulator of globin gene HBG1), which was found to be significant both in the GSMR analysis and also in the COVID-19 host genetics update, are regulators of the foetal haemoglobin (HbF). HBG1 and HBG2 which constitute the gamma chains of HbF are normally expressed in foetal liver, spleen and bone marrow. HbF is slowly converted into adult haemoglobin (HbA) during the initial months of a baby’s development^32^. Based on these observations we postulate that in contrast to adults, BCL11A through its regulation of HbF could possibly have a protective effect in neonates and babies, who were relatively less affected by severe COVID-19. Of note, normal serum iron levels in the blood is maximum in newborns (100–250 µg/dL) followed by adult male (65–176 micro µg/dL/dL), adult females (50–170 µg/dL/dL) and children (50–120 micro µg/dL/dL) (source url https://en.wikipedia.org/wiki/Serum_iron*).* Further, iron, which is an integral part of HbF, is also a common element in the severe COVID-19 related gene CYP4B1^29^ and LCN2^33^. Continuing on the theme of haematology, we highlight two other genes from our severity list namely (1) HBBP1 is a gene involved in erythropoiesis^34^ and (2) NFIX, a gene involved in life long sustenance of vertebrate blood^35^. NFIX (also known as CCAAT-binding transcription factor) has a dual role of acting as a RNA polymerase II promoter for vertebrate globin genes^36^ and also acts as an initiation factor for adenovirus DNA replication. The NFIX CCAAT box transcription factor is also involved in oxidative stress response^37^ during muscle regeneration by macrophages. It also interacts with a non heme iron-binding protein Pirin (PIR) which in turn is regulated by the antioxidant response gene NRF2^38,39^. Other genes from our severity list include SLC22A31, a gene responsible for organic anion transport across the cell membrane and SLC6A20, a gene involved in Na (+)- and Cl (-)-dependent uptake of amino acids such as L-proline. SLC22A31 is a member of the conserved OAT family of solute carriers^40,41^ which also has a closely related member gene SLC22A17 (not reported to be associated with COVID-19 severity so far). SLC22A17^42^ is known for regulating LCN2, an iron trafficking gene. LCN2 was recently shown to be important in neuroinflammation^43^ and also separately reported to be of significance by the COvid-19 Multi-omics Blood Atlas (COMBAT) study^33^. In the COMBAT study LCN2 was one of the main genes separating COVID-19 severity groups of patients from sepsis. Further, a recent study^44^ demonstrated that iron oxide nanoparticles (IONPs) iron oxyhydroxide (IOHNPs) impairs SARS-CoV-2 virus in vitro and further induces expression of LCN2 (seven-fold), SLC11A2 (encoding divalent metal transporter 1 (DMT1) also known as Natural resistance-associated macrophage protein 2 (NRAMP 2)) (three-fold) and SLC40A1 (FPN1) (38-fold). IONPs have been shown to induce ROS and oxidative stress in different cell types including macrophages^45,46^.

Additionally, from the COVID-19 update study we highlight ATP5PO, a gene which is involved in proton conductance in the mitochondrial matrix and ATP11A which is involved in influx of Ca^2+^ into the cells. In normal human physiology, due to the toxic nature of iron and ROS, these entities are tightly regulated within the cell and in the body. The daily dietary requirement for iron is only one milligram whereas the rest of the requirement of two to three milligrams of iron is recycled inside the body from older cells, mainly through dying red blood cells (RBCs). Macrophages are known to be one of the main cell types responsible for iron recycling from RBCs and they can provide up to 75% of the total requirement^47^. Erythropoiesis or RBCs generation is a highly dynamic process, and they are produced within the bone marrow at rates exceeding 2 million new cells per second. To this end, we highlight that red-blood-cell count was found to be the most significant protective trait against COVID-19 severity as depicted in Fig. 3 of the mendelian randomisation analysis of COVID-19 host genetics study^30^. Compared to most cells, RBCs have a short life span of around 120 days. RBCs undergo de-nucleation during their initial development and inherently lack mitochondria due to which they are not able to metabolise fatty acids, hence solely rely on glycolysis for energy (ATP) production. This observation perhaps explains the importance of SLC6A5, a gene responsible for the glycolysis and fructose transport from our severity list^48^. The hypothesis that RBC lifespan gets shortened or disrupted in conditions such as sepsis or severe COVID-19 perhaps warrants further investigation. In humans, macrophages first develop during embryogenesis and subsequent to the yolk-sac phase of embryo development, these cells get distributed to major organs where they differentiate and develop further as resident macrophages^49,50^. During their lifetime, the resident macrophages maintain tissue homeostasis and recycle iron and other metabolites from the tissue microenvironment^51^. Compared to the blood monocyte derived non-resident macrophages, the resident macrophages self-renew and are relatively long lived. These observations are of added importance for COVID-19 since results published in a immunological study between adults and children demonstrates that the interaction of resident and non-resident macrophages plays a key role in protecting young children against COVID-19^26,52,53^.

Oxygen is the third most abundant element on the earth’s crust followed by other metals including iron (the most abundant transition earth metal) magnesium, cobalt, nickel etc. It has been reported that 10 to 15% of human genes in the cell are switched on and off by oxygen availability, which in turn affects important cellular functions like the tricarboxylic acid (TCA) cycle, DNA replication, cell division or cell cycle arrest^54^. Of note, in COVID-19 severe cases as well as in sepsis, multiple studies have shown that oxygen supply through ventilation is of critical importance for palliative care. We have already discussed the importance of iron for COVID-19 severity, here, in addition, we highlight the putative role of Magnesium in COVID-19. Magnesium like iron is a divalent cation and is part of more than 300 enzymes in the human body and forms an important part of protein translation machinery^55^ and protects the mitochondria from oxidative stress^56^. Magnesium is also an integral part of biochemistry of GTpase, guanine nucleotide exchange factors (GEF) and GTPase-activating proteins (GAP)^57–59^. In this regard, we note several genes of interest from these classes of enzymes and molecules from the COVID-19 host genetics published results. Some examples include ARHGAP27, ARHGEF38, ARL17A and RAB2A (Table 1, **supplementary Table ****6**).

The fundamental role played by elements such as iron, oxygen, cations and anions in human cell biology or physiology^60^ could potentially run very deep, but rarely measured or characterised in current human sequencing studies^38^. The role of such elements in disease pathogenesis perhaps remains under explored at present^61^. Hence, it is possible that current modalities of sequencing DNA and RNA on its own may be inadequate to fully understand the full spectrum of COVID-19 manifestations seen in humans. The link between OAS1 and LTZFL1 through metabolomic signatures perhaps reflects cellular dynamics related to charge, flux and metabolite concentration (e.g., ROS). Many of these events occur in small time changes which are usually not considered in current investigations. For instance, iron trafficking can change intracellular pH for a time duration of only a few minutes. Therefore such dynamics^62^ are unlikely to be reflected through gene expression or genomic sequencing alone and might require new multimodal data integration approaches in future^63^. To conclude, based on our results and observations, here, we hypothesise that iron trafficking, macrophage biology and ROS might have an important role to play in the pathogenesis of severe COVID-19 (Table 1, **supplementary Table ****6**).

### Limitations

The results presented in this study should be interpreted while considering some of the limitations. Firstly, we echo that CNV genotyping and breakpoint ascertainment from microarray platforms remain viable but challenging. For instance, our study has limited probe coverage across the genome with only 10,764 common probes for NFBC 1966 and NFBC 1986. We also note that differences in the underlying genotyping platforms in NFBC cohorts could have led to some of the technical biases which is hard to ascertain and correct for at present. The NFBC 1986 cohort was genotyped on the Illumina Cardio-MetaboChip which is known to be enriched for probes related to lipids, cardiometabolic and anthropometric traits. Further, we note that despite having the same CNV pre-processing and analysis pipeline the inflation factors in NFBC 1986 cohort are lower in general compared to the NFBC 1966 cohort (**Supplementary Table ****3**). Here, the use of Cardio-MetaboChip in NBFC 1986 could a possible reason for this difference. We further note that the NFBC 1966 subjects were recruited two decades earlier in the 1960s where it is possible that due to a different era, the NFBC 1966 subjects were exposed to a different environment (medication use, lifestyle etc.). All these factors could have contributed to the manifestation of slightly different metabolic profiles between the two cohorts. However, this hypothesis remains to be validated and explored further. Next, regarding some of the limitations of our CNV analysis, we highlight that the dosage effect of CNVs were used in an additive model setting where the combined effect of deletions and duplication were analysed. A stratified analysis of deletions and duplications is left as future work (**Supplementary Figs. 4**,** 5**). Also, our CNV analysis pipeline is targeted at elucidating the role of small to intermediate length CNVs and does not address rare variants. Lastly the COVID-19 results and biology should be analysed by considering the above-mentioned limitations. The best use cases for our research work are new experiments and hypothesis generation and we advise against any new clinical applications without proper scientific validation. We also note that at present there is limited scientific literature on the topic of genetics of lipid peroxidation. We hope our study will lead to follow up experiment on lipid peroxidation and further elucidate its role in inflammation and disease severity. To this end our univariate and multivariate metabolomic signatures for the most relevant COVID-19 genes can prove to be a useful resource.

## Methods

The Northern Finland Birth Cohort 1966 is a longitudinal cohort from Northern Finland consisting of children (*n* = 12,068) born in the provinces of Oulu and Lapland, Finland. All subjects in this cohort were recruited between 1 January 1966 and 31 December 1966. The NFBC 1986 cohort study (*n* = 9,432) is a subsequent study to NFBC 1966, where subjects were recruited from the same area as NFBC 1966 but between the period 1 July 1985 and 30 June 1986. Briefly, all study subjects in NFBC 1986 and NFBC 1966 were monitored from pregnancy to adolescence and blood samples were collected from study participants at 31 years of age. Blood samples were drawn after overnight fasting and subsequently stored in -80 °C. Next, high-throughput metabolomic measurements, including deep lipoprotein subclass characterisation were made by NMR platform (Bruker AVANCE III spectrometer operating at 500 MHz) developed by Nightingale Healthcare Limited. Further details about metabolite particle sizes and ratios have been reported in earlier studies^11^ (**Supplementary Table ****2**). Only one metabolite (HDL3_C, Total cholesterol in HDL3) displayed technical variation between NFBC 1966 and NFBC 1986. This variation was subsequently corrected in a new batch of metabolomic measurements which was subsequently used for all our analyses (**Supplementary Figs. 6**,** 7**).

NFBC 1986 and NFBC 1966 were genotyped on Illumina CardioMetaboChip and on Illumina Human370CNV-Duo chip at the Broad institute. Based on genomic location (hg19) there was concordance in 10,764 of the genotyping probes between the two genotyping platforms thus enabling pooled and meta-analysis for common probes across these two cohorts.

All methods and experimental procedures for NFBC cohorts were carried out with adherence to regulations and recommended guidelines. Both studies and experimental protocols have been approved by the regional ethics committee of University of Oulu and the Northern Ostrobothmia Hospital. Informed consent in writing was obtained from each participant’s legal guardian for NFBC 1966 and NFBC 1986 cohorts.

### Data normalisation and CNV calling

All methods and software used in this study are based on an earlier reported pilot study^8^ where we characterised common CNVs in the TSPAN8 gene. Briefly, prior to CNV calling, as data normalisation procedure we corrected for genomic wave effects by applying a localised loess function in a 500 kb window and corrected total intensity measurement -r log R ratio (LRR) for GC content. Next, we used the cnvHap algorithm^64^ in its population aware mode with Illumina platform emission parameters to detect and genotype CNVs in NFBC 1986 and NFBC 1966. cnvHap integrates log R ratio (LRR) and B Allele Frequency (BAF) into a single CNV-SNP haplotype Hidden Markov Model for detecting and genotyping CNVs. The underlying model which best describes our CNV analysis is a mirror model where we try to capture the combined additive effect of deletions, duplications, and copy-neutral states into a single CNV genotype vector. Briefly, we ran the cnvHap hidden Markov model (HMM) with four hidden states i.e., state 0 for homozygous deletions, state 1 for heterozygous deletion, state 3 for heterozygous duplication and state 4 homozygous duplication. Next the cnvHap algorithm derives the posterior probability of each of these four hidden states and for every genotyping probe it calculates expected CNV genotypes. For example, if at a particular probe a sample has CNV genotype assigned as 1 (heterozygous deletion) with probability of 0.7 then the expected CNV genotype is calculated as: 1*0.7 + 2*0.3 = 1.3. A final CNV genotype, referred to as “*countAll*” in the cnvHap output was calculated and used for all our analyses. The countAll value ranges from 0 to 4 and is derived by summing the expected CNV genotype values across deletion, copy-neutral and duplication states. Of note, apart from *countAll*, cnvHap also produces two other vectors of expected CNV genotypes namely (1) *state.0 or* deletion-only model and (2) *state.2 or* duplication-only model. Here, we have only analysed and reported results for the countAll values. Further, to disentangle the effect of deletions and duplications at the same locus (while using countAll values) we advise the following. One can stratify the countAll CNV genotypes into deletions (e.g., samples with countAll values < 1.75), copy-normal and duplications (countAll > 2.5). Next, based on this sample stratification one can apply non-parametric tests like Mann-Whitney to calculate significance of phenotypic distributions. (**Supplementary Figs. 4**,** 5**)

### Association analysis

We next applied both univariate and multivariate models for associating expected CNV genotypes with 228 metabolomic phenotypes using the Multiphen R package^65^. The Multiphen multivariate model uses reverse regression, where the outcome is expected CNV genotypes and predictors are metabolomic measurements. As described in our earlier study^8^, a metabolomic signature is defined as a unique set of uncorrelated metabolites obtained on application of backward variable selection to the joint model of the Multiphen method. In all cases a robust set of covariates were used. This included 50 LRR principal components (PCs) and gender. All univariate and multivariate metabolomic signatures for CNV genotypes were independently validated (without CNV calling) by using LRR based association model with LRR 50 principal components and gender as covariates. To correct for multiple testing in our results we applied an alternate method to Bonferroni correction referred to as modified Sidak-Nyholt correction. Like our previous study^8^, here the net effective number of tests considering the high degree of correlation is determined by applying the following correction, where lambda is the eigen decomposition of the correlation matrix of metabolomic phenotypes. All reported p-values in the manuscript have been corrected for multiple testing using the following approach.

$${{\text{M}}_{{\text{effective}}}}=1+({\text{M}} - 1)(1 - {\text{Var}}({\lambda _{{\text{observed}}}})/{\text{M}})$$

SNP association results are nominal and meant only for replication with gender used as covariate (Genotype PCs not available). No SNP imputation was performed; hence results are restricted to only the probes of the genotyping platform.

### COVID-19 GWAS annotations

The GRASP database lists GWAS results for COVID-19 susceptibility from various studies including from the UK Biobank (UKBB)^66^. GWAS in the UKBB studies were performed with SAIGE software (v0.38) with appropriate control for population stratification, relatedness and case-control imbalance, and further adjusted for age, sex, and 10 genetic principal components. Variants were further filtered on imputation quality (r^2^ > 0.3), minor allele count (> 2), and minor allele frequency (MAF > 0.0001). Each release of UKBB consisted of up to 65 GWAS further divided into the following groups (1) COVID-19 susceptibility (cases, *n* = 18,481) (2) COVID-19 hospitalisation (*n* = 3,260) (3) Severe COVID-19 with respiratory failure (*n* = 1,244) and (4) COVID-19 related death (*n* = 1,104). GWAS on data releases were further stratified by sex, ancestry, and trans-ethnic groups namely 459,250 of European ancestry (EUR), 7,644 of African ancestry (AFR), 9,417 of South Asian ancestry (SAS), and 11,009 of other ancestries (Other). Here, based on genomic location (hg19) we juxtaposed results from these GWAS (*n* = 612) to the metabolomic signatures in NFBC 1966 and NFBC 1986 in a variant (CNV or SNP) agnostic way. A complete list of study groups is provided in **supplementary Table ****4**.

### Multi-omic annotation database for metabolomic signatures

To gain further biological insights, we have developed a metabolomic signature database with detailed multi-omic annotations for all our signatures and CNV maps in NFBC 1986 and NFBC 1966 (**Supplementary Table ****1**). The database consists of a beacon table, where all loci are cross referenced with metabolomic signatures, CNVs and multiple other annotations collated from public databases, including annotations from the global lipids consortium database.

**Table 1:** Summary of COVID-19 hallmarks and the genes associated with them.

COVID-19 Hallmark	Genes
Haematological	ABO, BCL11A, FUT2, GATA2, ICAM4, LZTFL1, PLSCR1, SLC22A5, THBS3, TCF7L2
Divalent cations Fe^2+^/Mg^2+^/Ca^2+^	ANXA11, CIB4, CYP4B1, FDX2, MBL1P, NUCB1, PIR, SLC39A8
GTpase/GEF/GAP family, Protein translation	ARHGAP27, ARHGEF38, ARL17A, CEP97, EIF5AL1, LZTFL1, PLEKHM1, RAB2A, RASIP1
Voltage-gated ion transport, Mitochondria, ROS, Neurological	ACSF3, ATP11A, ATP5PO, ATP6V1G2, CAT, FOXG1, HCN3, KCNC3, MRPS6, MTX1, NSF, RPL24, SLC22A31, SLC34A2, SLC6A20, TOMM7, TRIMM46, TTC10
Macrophage biology	CCR1, CCR2, CCR3, CCR5, CSF3, ICAM3, IRF1, IRF7, NR1H2

Genes listed above have been reported to have significant association with COVID-19 either from the GWAS of COVID-19 host genetics initiative study or the study involving GWAS of COVID-19 severity groups (including GSMR analysis) (Supplementary Table 6).

## Electronic supplementary material

Below is the link to the electronic supplementary material.

Supplementary Material 1

Supplementary Material 2

Supplementary Material 3

Supplementary Material 4

Supplementary Material 5

Supplementary Material 6

Supplementary Material 7

## Data Availability

Further information and requests for resources and reagents should be directed to and will be fulfilled by the lead contact, Dr Tisham De (tisham.de08@imperial.ac.uk or de.tisham@gmail.com). NFBC participant data are available to scientific researchers with appropriate access permissions. Further details are available here https://www.oulu.fi/nfbc/materialrequest. We have designed and developed a custom MySQL database for all association results reported in this study including summary statistics of univariate and multivariate metabolomic signatures for CNVs and SNPs. These include allele frequency of the variant (CNV or SNP), beta and P values from association methods, sample size and cohort names. All above mentioned univariate and multivariate signatures have been further annotated with publicly available multi omics annotations for wider biological context and understanding. A summary of the various database tables and their associated annotation is described in supplementary Table 1. This custom database of metabolomic signatures is available for public download from the Zenodo scientific repository. DOI 10.5281/zenodo.12737754 Url 10.5281/zenodo.12737754. Another important component of this database includes COVID-19 GWAS results made available for public download from National Heart, Lung and Blood Institute (NIH, US Government). The public download link of COVID-19 GWAS results that we used is: https://grasp.nhlbi.nih.gov/Covid19GWASResults.aspx. The associated publication for this dataset is available here: https://www.cell.com/hgg-advances/fulltext/S2666-2477(22)00011-2. All data in our database are publicly available or open source hence do not require any ethical guidelines.

## Code availability

All codes used to process and analyse data are published, and the source code is currently available at. Multiphen: https://github.com/lachlancoin/MultiPhen. cnvHap: https://www.imperial.ac.uk/people/l.coin. cnvHitSeq: https://sourceforge.net/projects/cnvhitseq. This study did not generate new reagents or software code.

## Material availability

NFBC material can be requested from the consortium website https://www.oulu.fi/nfbc/materialrequest.
