# Supplementary material for: Plasma metabolomic signatures for copy number variants and COVID-19 risk loci in Northern Finland populations
Source: Sci Rep. 2025 Apr 16;15:13172. doi: 10.1038/s41598-025-94839-9 (PMC12003712; doi:10.1038/s41598-025-94839-9)
Supplement: Supplementary file 1 — Supplementary Material 1 [file 41598_2025_94839_MOESM1_ESM.pdf]

Supplementary material

Plasma metabolomic signatures for copy number variants and COVID-19 risk loci in Northern Finland Populations

Tisham De[1,2,3], Lachlan Coin[3,4], Jethro Herberg[3], Michael R Johnson[5], Marjo-Riitta Järvelin[6,7,8,9,10]

1. **Department of Epidemiology and Biostatistics, School of Public Health, Imperial College, London, UK**
2. **Department of Genomics of Common Diseases, Imperial College London, UK**
3. **Department of Infectious Disease, Imperial College London, UK**
4. **Department of Microbiology and Immunology, University of Melbourne at The Peter Doherty, Institute for Infection and Immunity, Melbourne, Australia**
5. **Department of Brain Sciences, Imperial College London, UK**
6. **Centre for Life Course Health Research, Faculty of Medicine, University of Oulu, Oulu, Finland**
7. **Unit of Primary Health Care and Medical Research Center, Oulu University Hospital, Oulu, Finland**
8. **Department of Epidemiology and Biostatistics, Medical Research Council–Public Health England**
9. **Centre for Environment and Health, Imperial College London, London, UK**
10. **Biocenter Oulu, University of Oulu, Oulu, Finland**

Index

1) Supplementary figures
2) Supplementary table legends
3) Data sources and urls

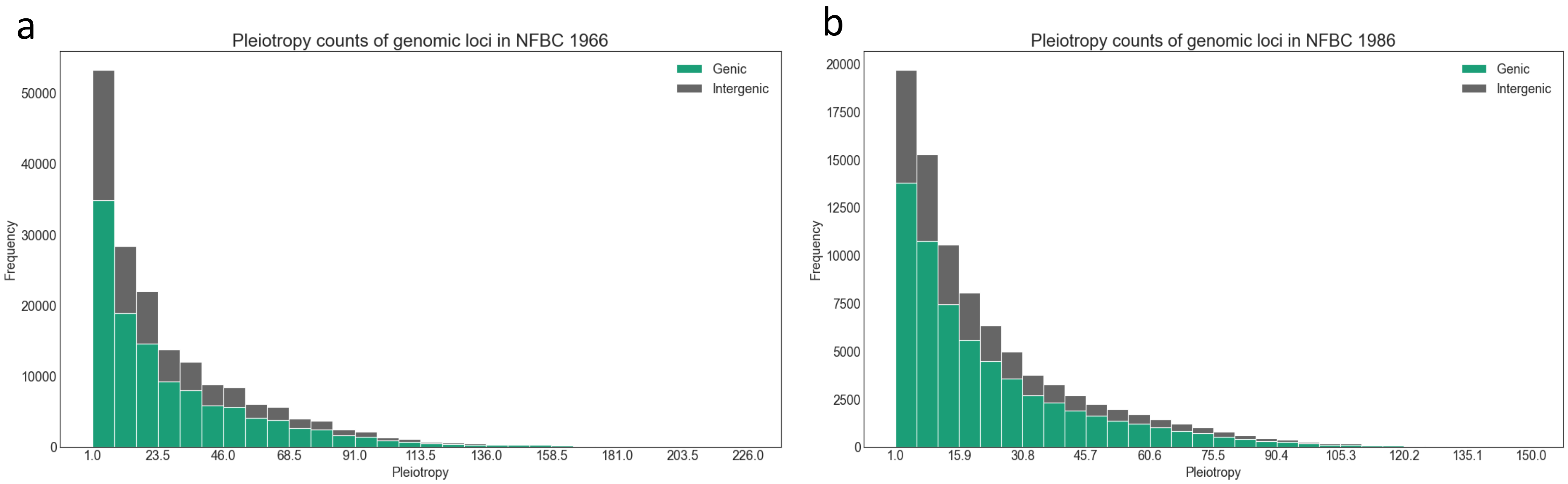

Supplementary figure 1. CNV Pleiotropy. Pleiotropy counts of CNV loci in NFBC cohorts. a) NFBC 1966 CNV genotypes. b) NFBC 1986 CNV genotypes.

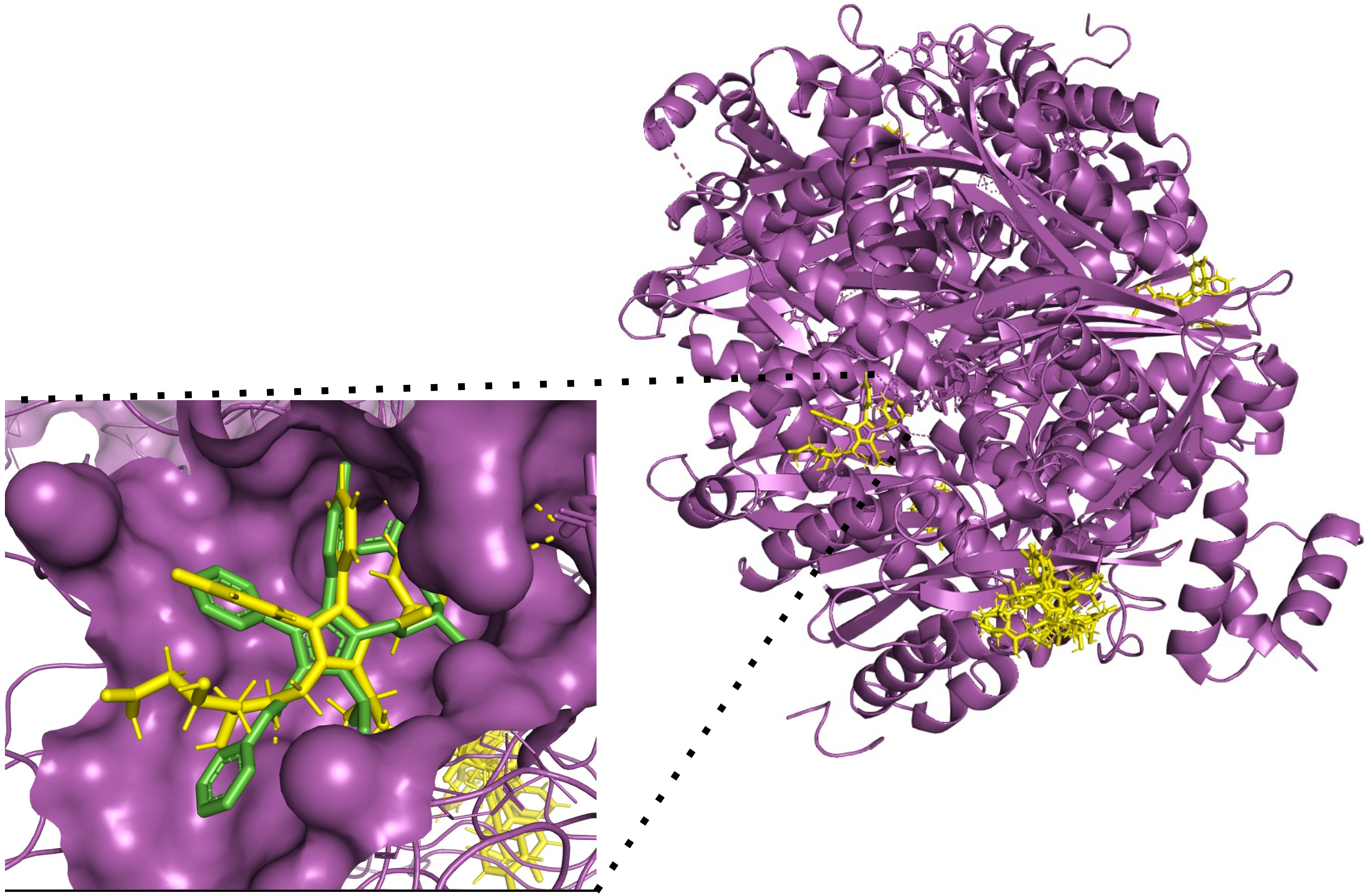

Supplementary figure 2. Figure depicting molecular docking of atorvastatin with the catalytic domain of Human HMG-COA reductase (PDB id: **1HWK**). Docking was done using the AutoDock Vina algorithm which produced nine different binding poses for atorvastatin. In the zoomed section of the figure, experimentally derived pose of atorvastatin marked in green can be compared to the AutoDock Vina predicted pose marked in yellow. Binding affinity for this particular pose was -6.4 kcal/mol with RMSD=33.917. Only one out nine different predicted poses from AutoDock Vina matched with the experimental results. The binding affinity for the nine different poses from AutoDock Vina were -6.6, -6.5, -6.4, -6.4, -6.4, -6.3, -6.3, -6.2, -6.1 kcal/mol with RMSD of 0, 50.262, 49.472, 2.279, 33.917, 38.113, 50.292, 48.548, 49.444 respectively.

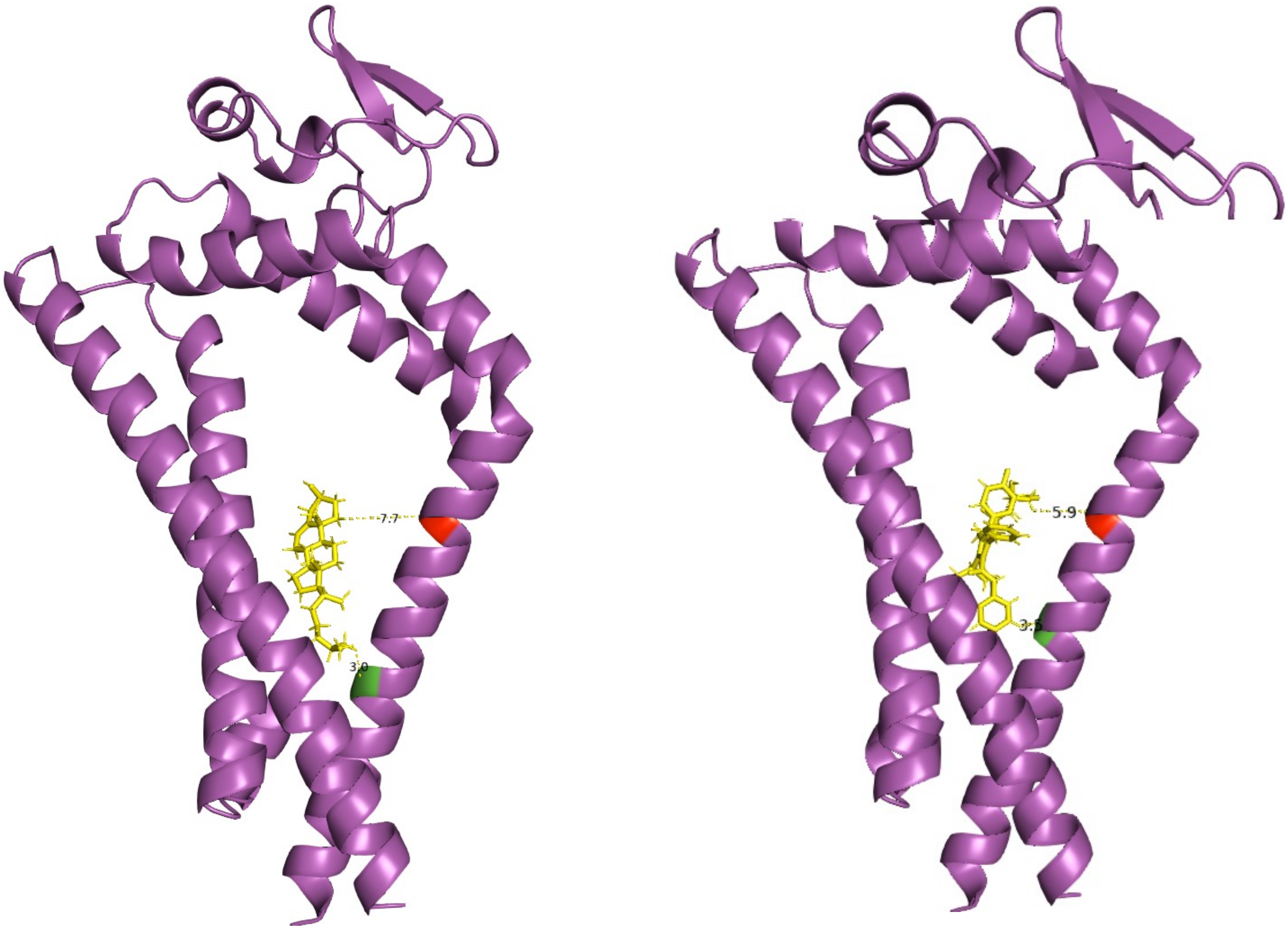

Supplementary figure 3. TSPAN8 CNVs was shown to have pleiotropy of up to 61% (22/36) for LDL and its subclasses in NFBC cohorts[1]. Left panel shows molecular docking of TSPAN8 protein structure (generated by AlphaFold 2 algorithm) with cholesterol using the AutoDock vina method. Right panel shows molecular docking of TSPAN8 protein structure with atorvastatin with similar molecular distances as cholesterol.

1. De, T., Goncalves, A., Speed, D., Froguel, P., Gaffney, D.J., Johnson, M.R., Jarvelin, M.R. and Coin, L.J., 2021. Signatures of TSPAN8 variants associated with human metabolic regulation and diseases. *Iscience*, *24*(8).

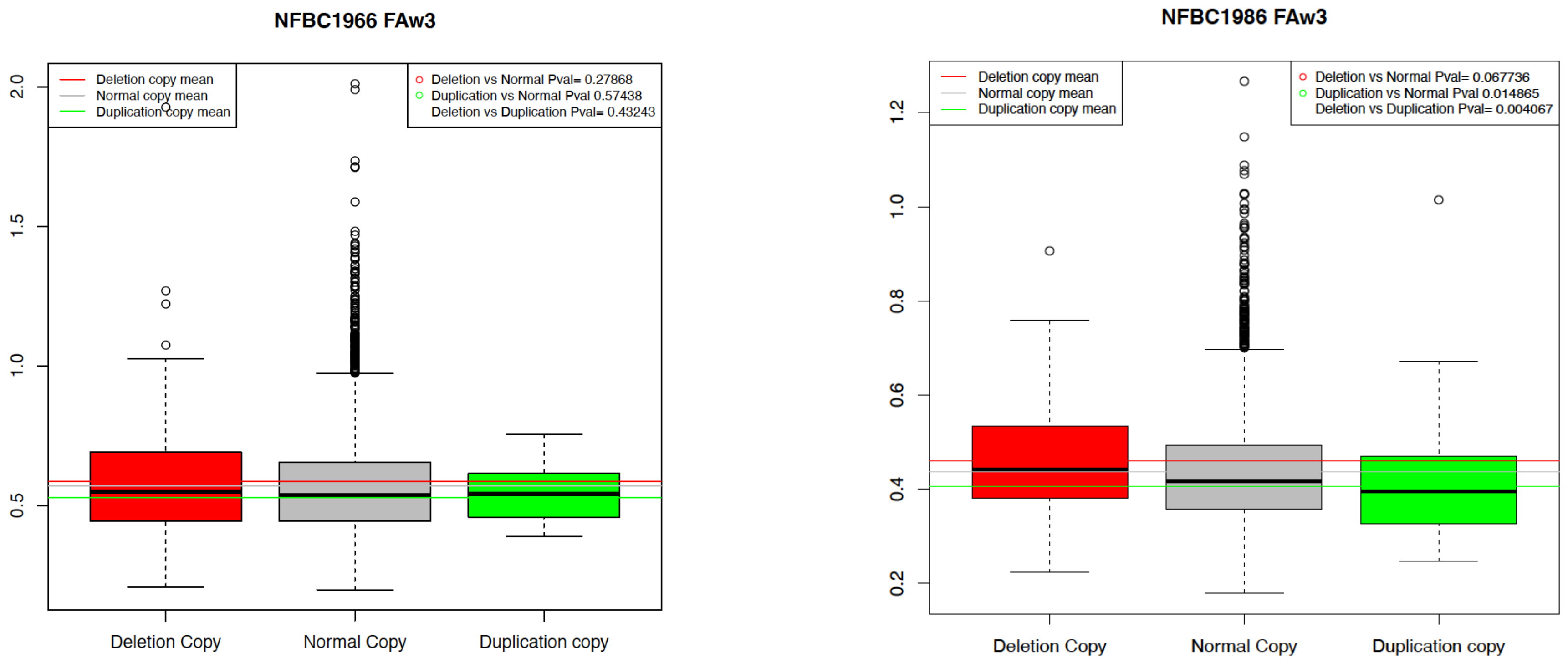

Supplementary figure 4. Box plot showing the stratified effect of CNV at chr12:71523134 (TSPAN8 gene) for the Omega-3 fatty acids (FAw3) metabolite in NFBC 1986 and NFBC 1966. This is a preview of future work where a similar analysis for the whole genome in NFBC cohorts is envisaged.

Phenotype	Del vs Normal Pval	Dup vs Normal Pva	Del vs Dup I	Mean Deletic	Mean Norma	Mean Dup	Del > Norma	Dup > Norm	Del > Dup
AcAce	3.88E-01	8.05E-01	6.86E-01	0.04816884	0.0518519	0.05503642	FALSE	TRUE	FALSE
Ace	3.21E-01	8.87E-01	3.62E-01	0.04773507	0.04634864	0.04620926	TRUE	FALSE	TRUE
Ala	8.27E-02	8.31E-01	1.58E-01	0.43236522	0.41239992	0.41042526	TRUE	FALSE	TRUE
Alb	5.89E-01	2.73E-01	2.79E-01	0.10473	0.10320847	0.103242	TRUE	TRUE	TRUE
ApoA1	8.27E-02	9.44E-01	2.47E-01	1.54889855	1.51925684	1.52373684	TRUE	TRUE	TRUE
ApoB	7.45E-03	6.59E-02	1.67E-03	0.89541304	0.83870764	0.80685158	TRUE	FALSE	TRUE
ApoB_ApoA1	2.47E-02	3.09E-02	1.52E-03	0.57752754	0.55671073	0.53459579	TRUE	FALSE	TRUE
bOHBut	6.90E-01	2.28E-01	2.57E-01	0.13757899	0.14632339	0.16166295	FALSE	TRUE	FALSE
Cit	1.52E-03	3.54E-01	6.90E-02	0.09558203	0.10449725	0.10155947	FALSE	FALSE	FALSE
Crea	8.28E-01	8.10E-01	9.63E-01	0.05550072	0.05598555	0.05594463	FALSE	FALSE	FALSE
DHA	1.07E-01	2.67E-02	8.22E-03	0.15145464	0.14254406	0.13228611	TRUE	FALSE	TRUE
DHA_FA	8.71E-01	1.85E-02	6.47E-02	1.29494348	1.29692217	1.23860947	FALSE	FALSE	TRUE
EstC	5.52E-03	3.99E-01	7.52E-03	3.34931884	3.13180235	3.06521053	TRUE	FALSE	TRUE
FAw3	6.77E-02	1.49E-02	4.07E-03	0.46101884	0.43698425	0.40684105	TRUE	FALSE	TRUE
FAw3_FA	9.49E-01	1.99E-02	1.33E-01	3.96449275	4.00201101	3.83154737	FALSE	FALSE	TRUE
FAw6	1.26E-02	4.46E-01	1.50E-02	3.73495652	3.5413446	3.48538947	TRUE	FALSE	TRUE
FAw6_FA	1.56E-01	1.49E-01	4.49E-02	32.215942	32.5272555	32.8624211	FALSE	TRUE	FALSE

Supplementary figure 5. A stratified analysis of deletions and duplications at chr12:71523134 (TSPAN8 gene) and their effect on NFBC metabolites. This is a preview of future work where a similar analysis for the whole genome in NFBC cohorts is envisaged.

a

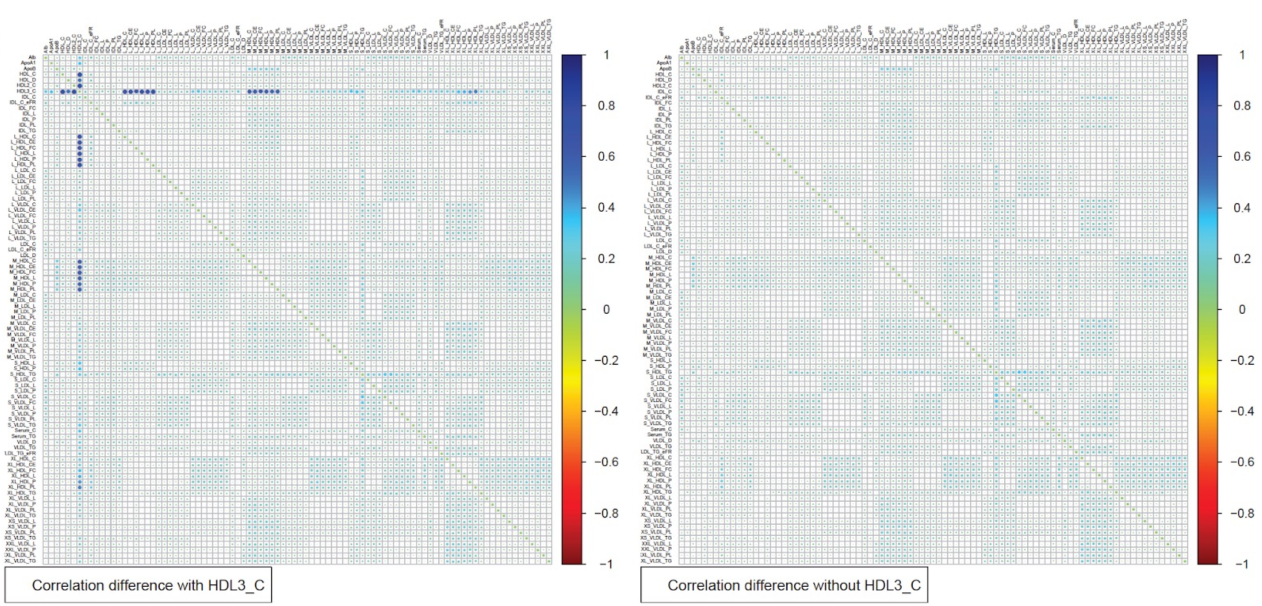

b

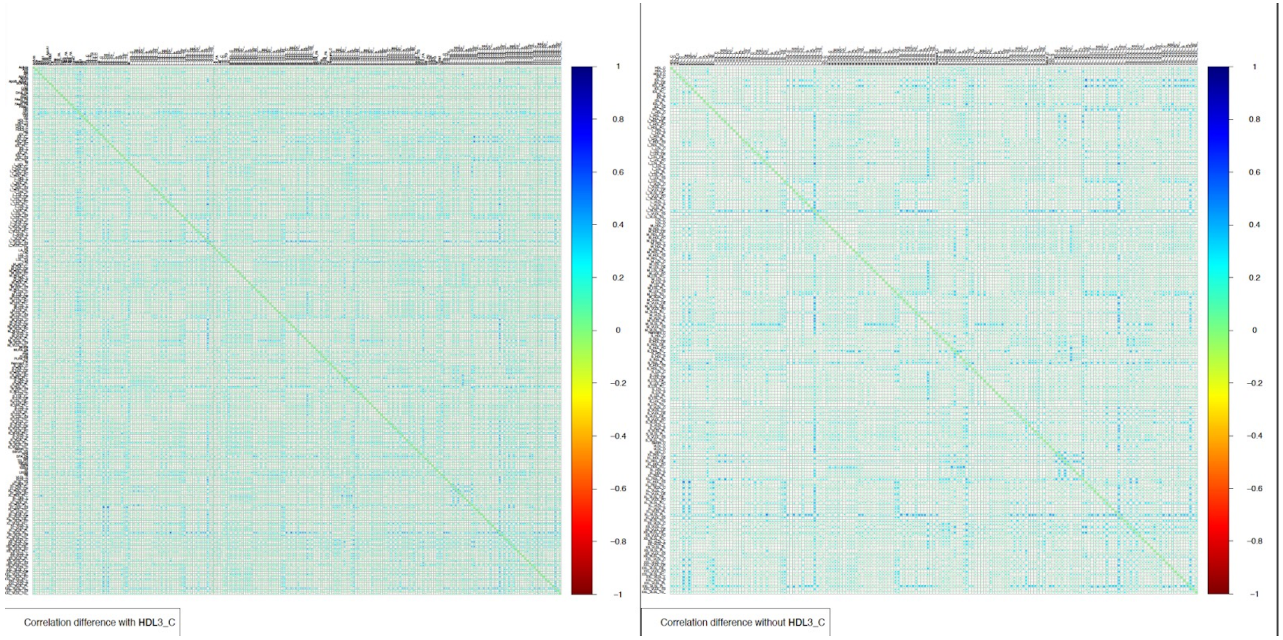

Supplementary figure 6. Batch effects in metabolomic measurements. Heatmap of absolute difference in correlation matrix for 228 metabolites in NFBC 1986 and NFBC 1966 across two batches of metabolomic measurements: a) 2012 and b) 2015

De, T., Goncalves, A., Speed, D., Froguel, P., Gaffney, D.J., Johnson, M.R., Jarvelin, M.R. and Coin, L.J., 2021. Signatures of TSPAN8 variants associated with human metabolic regulation and diseases. *Iscience*, *24*(8).

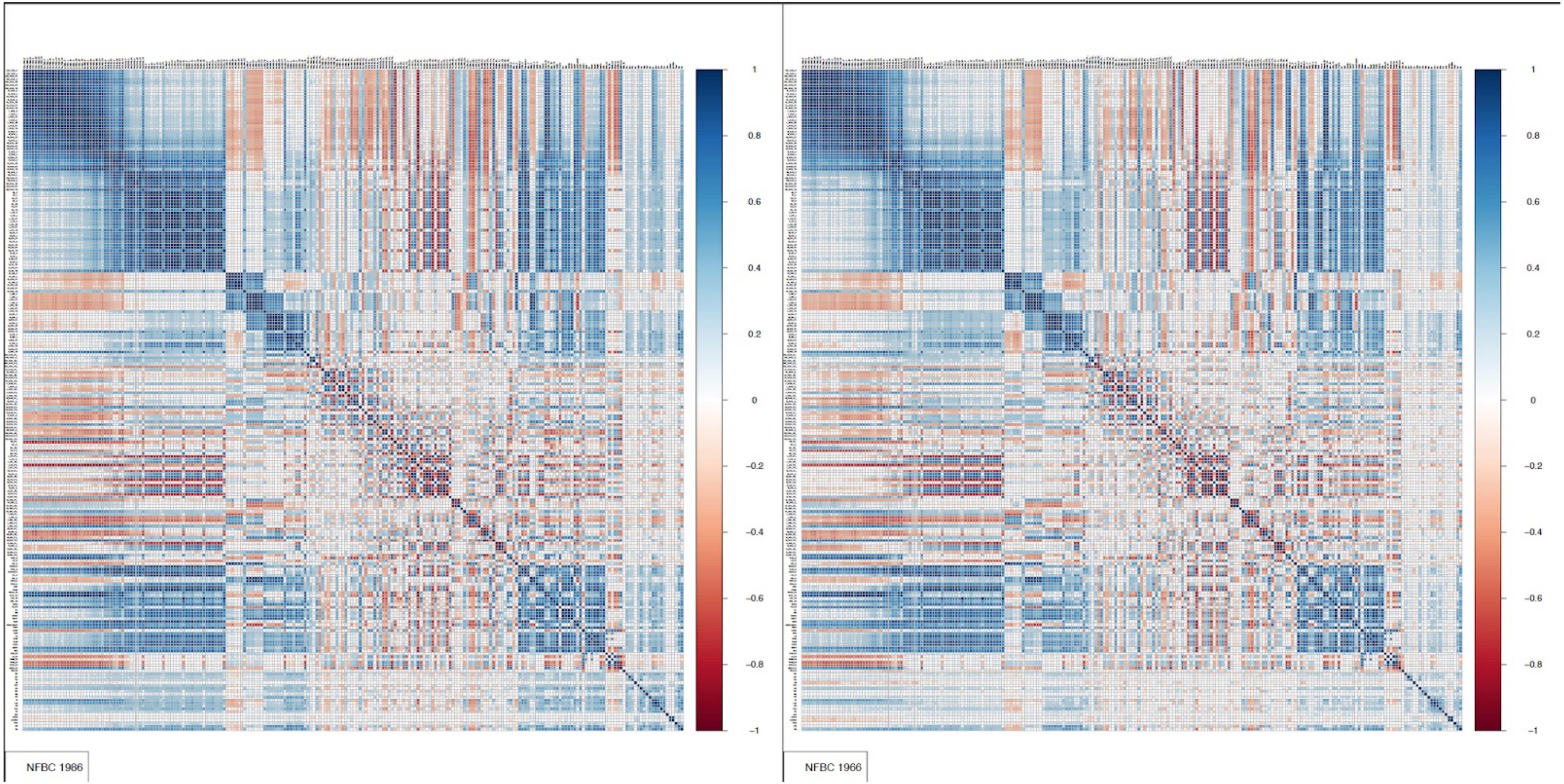

Supplementary figure 7. Heatmap showing correlation matrix of 228 metabolites in NFBC 1986 and NFBC 1966 cohorts from the 2015 batch of measurements (see supplementary figure 6).

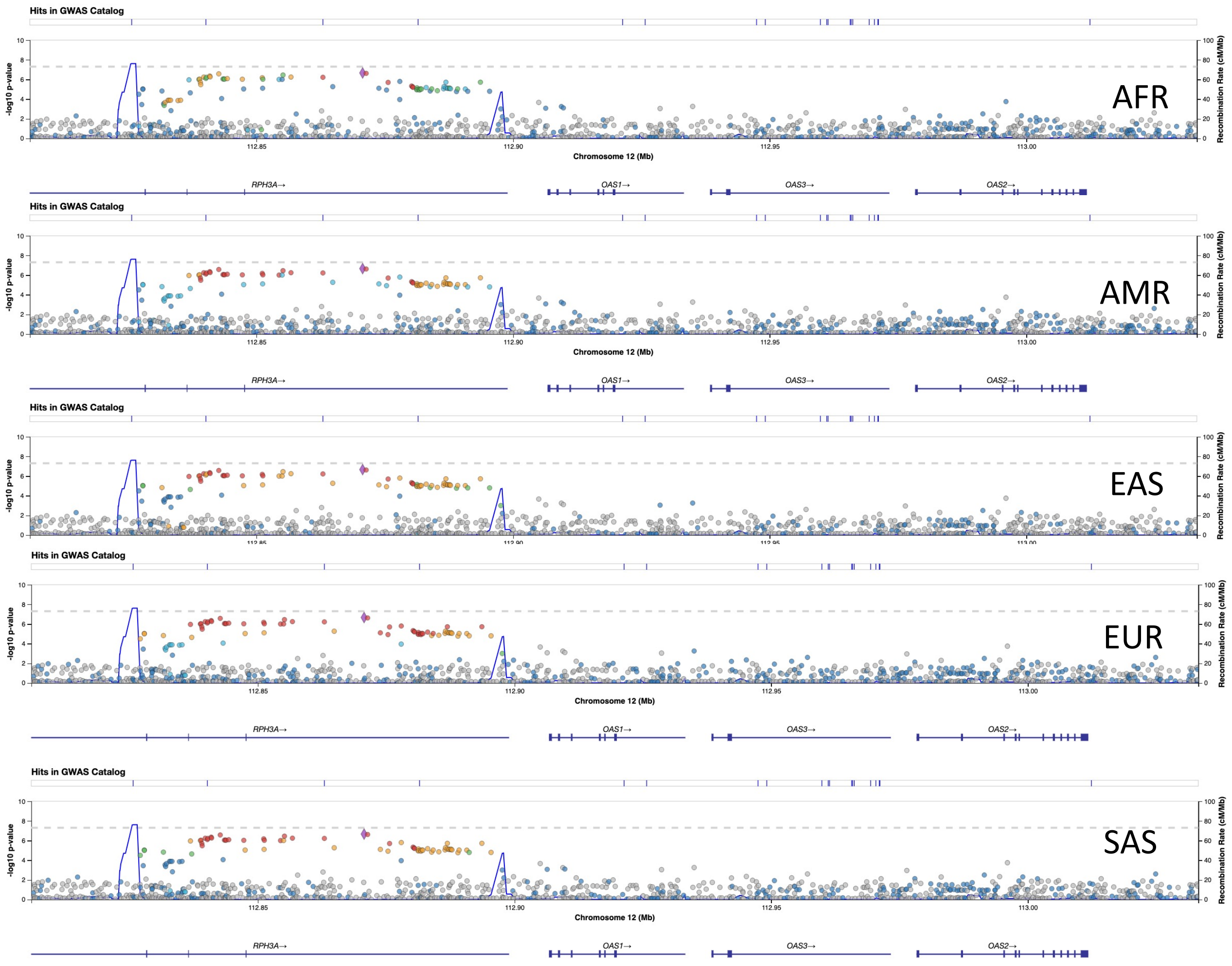

Supplementary figure 8. LD in OAS1. Linkage disequilibrium plot stratified by ancestry for OAS1 gene cluster in Topmed Phewas database for UK biobank (url https://pheweb.org/UKB-TOPMed/).

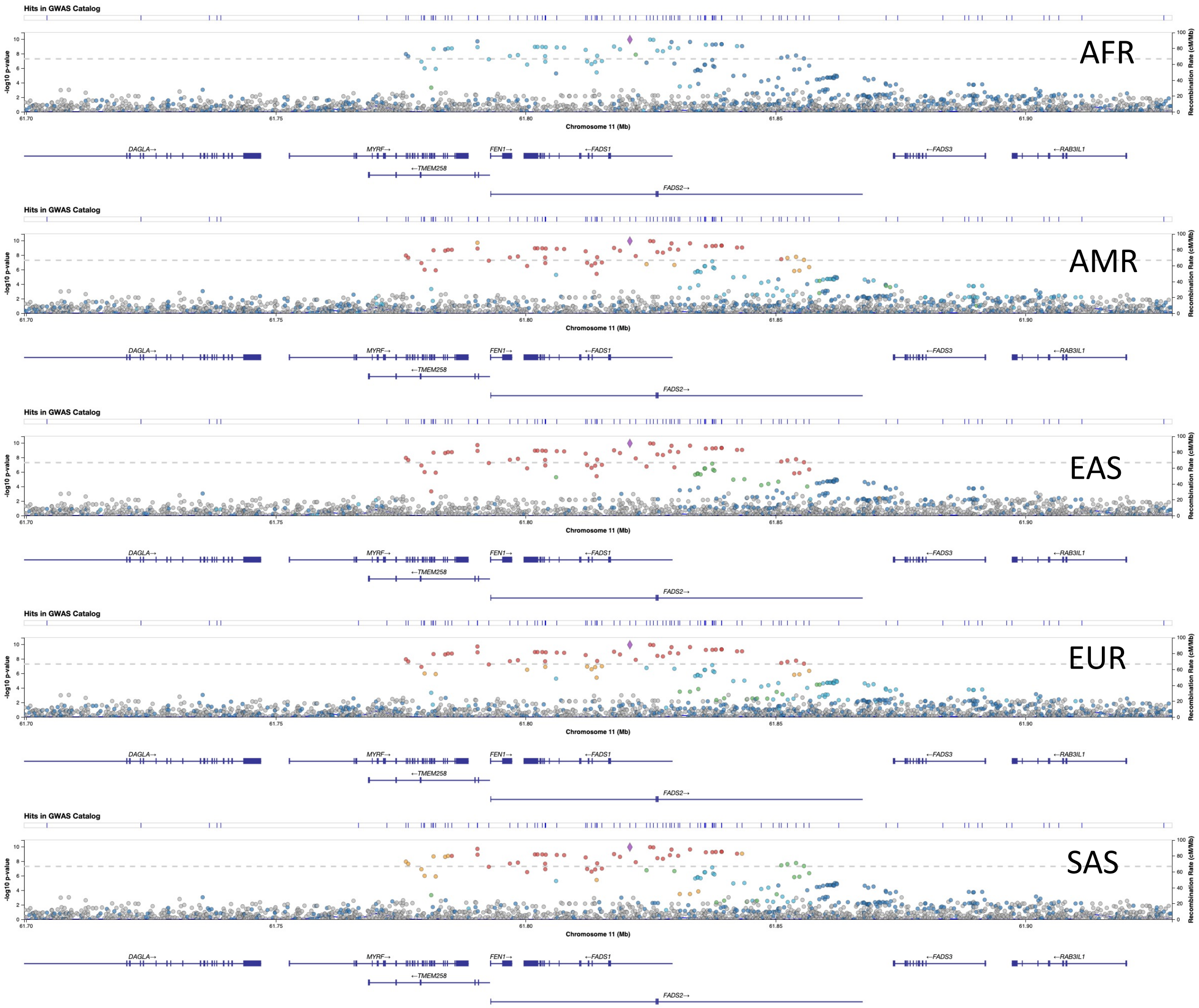

Supplementary figure 9. LD in FADS1/FADS2. Linkage disequilibrium plot stratified by ancestry for FADS1/FADS2 gene cluster in Topmed Phewas database.

Supplementary table legends

Supplementary table 1. Multi-omic database. Descriptions of the tables in the multi omic MySQL database for metabolomic signatures. Database is available at https://doi.org/10.5281/zenodo.12737754

Supplementary table 2. Metabolite names. Annotations for the NFBC metabolomic lipid names and their respective categories.

Supplementary table 3. CNV-metabolomic QTLs and inflation factors. CNV genotype association results and inflation factors.

Supplementary table 4. COVID-19 GWAS studies. Annotations for UK biobank COVID-19 GWAS study names.

Supplementary table 5. Signature comparison. Genome wide signature comparison of OAS1, LZTFL1, ACSL1 and NFIX.

Supplementary table 6. COVID-19 hallmarks. COVID-19 gene descriptions and the corresponding hallmarks

Data sources and urls

1. COVID 19 GWAS results from GRASP database: (Ref https://www.cell.com/hgg-advances/fulltext/S2666-2477(22)00011-2) https://grasp.nhlbi.nih.gov/Covid19GWASResults.aspx
2. Gene cards: https://www.genecards.org/
3. NFBC cohort details: https://www.oulu.fi/en/university/faculties-and-units/faculty-medicine/northern-finland-birth-cohorts-and-arctic-biobank/northern-finland-birth-cohorts
4. Nightingale metabolomics platform: https://nightingalehealth.com/technology
